# AI driven monitoring of orthodontic tooth movement using automated image analysis

**DOI:** 10.6026/973206300210173

**Published:** 2025-02-28

**Authors:** Chetan Dilip Patil, Aameer Fazluddin Parkar, Snehal Bhalerao, Pradeep Kawale, Anshuj Ajay Rao Thetay, Seema Lahoti

**Affiliations:** 1 Department of Orthodontics and Dentofacial Orthopedics, Yogita Dental College and hospital, Khed, Ratnagiri, Maharashtra, India; 2 Department of Orthodontics and Dentofacial Orthopedics, RKDF Dental College and Research Centre, Bhopal, Madhya Pradesh, India

**Keywords:** Artificial intelligence, orthodontic tooth movement, automated image analysis, deep learning, intraoral photographs, AI in orthodontics

## Abstract

Artificial intelligence (AI) driven automated image analysis accurately tracks orthodontic tooth movement by reducing reliance on time-consuming manual assessments. AI achieved 92% precision with a 0.25 mm error margin and a strong correlation (r = 0.94, p < 0.001) to manual measurements in a study of 100 patients. AI analysis took 3 seconds per image set, significantly faster than the 7-minute manual process (p < 0.001). Orthodontists rated AI reliability at 4.7/5, with 86% preferring AI-assisted monitoring. Thus, AI enhances treatment efficiency, standardization, and clinical decision-making.

## Background:

Orthodontic tooth movement (OTM) functions as a dynamic procedure which experiences influence from different biological along with mechanical elements. Orthodontic tooth movement tracking requires accurate evaluation because it supports effective treatment progress monitoring and allows for timely adjustments leading to superior results [1]. The current orthodontic measurement methods based on plaster models along with cephalometric radiographs and intraoral photographs take extensive time to process due to limited precision and variable human assessment quality [2, 3]. Medical science benefits from artificial intelligence technology to improve both diagnosis precision and treatment operations especially within the field of orthodontic practices [4]. Research also shows that Remote monitoring systems can be useful in times like the current pandemic to minimize in-person visits [5]. AI algorithms show outstanding performance when it comes to detecting dental anomalies and segmenting teeth and analysing cephalometric landmarks [6, 7]. Orthodontics utilizes AI for three applications which include treating outcome predictions, cephalometric tracing automation and facial growth assessments [8, 9]. Few studies in the available literature have studied how AI helps monitor orthodontic tooth movement through serial capture of intraoral images. The implementation of AI for automated image analysis represents an effective answer to traditional method constraints because it provides immediate results alongside unbiased quantitative measurements that can be repeated [10]. The field of artificial intelligence (AI) is currently experiencing an increase in popularity [11]. Therefore, it is of interest to evaluate automated image analysis using AI enhancement for orthodontic tooth movement tracking as compared to manual methods by measuring its accuracy together with time-effectiveness and reliability levels.

## Materials and Methods:

The forthcoming research enrolled one hundred patients who received fixed orthodontic teeth alignment therapy. The research selection included patients whose age ranged from 12 to 30 while lacking a medical history that affected tooth movement and presenting good oral hygiene practices. This study excluded patients who received previous orthodontic treatment together with those who had active orthodontic procedures at the same time. The research took place at one orthodontic clinic with the necessary ethical permissions granted to the study by the institutional review board. Every participant granted their consent for the study after being properly informed. Check-up pictures of the patient's mouth were recorded at five time points throughout six months at the first appointment and then again at four-week intervals. The study maintained standardized photographic protocols that established equal conditions between lighting and angulation and proper patient placement. The DSLR camera featured a macro lens and ring flash as part of its setup for image acquisition. The necessary plaster models of patient teeth were collected precisely during all measurement periods. Two experienced orthodontists used digital callipers to perform the manual measurements of tooth movement. Each measurement required double execution so the system recorded the mean value to achieve reliable results. A deep convolutional neural network (CNN) learned to process series of intraoral photographs so it could identify modifications in tooth positions. The AI system developed with Python and TensorFlow executed its training and validation processes using orthodontic images that practiced experts already labelled. The model used key landmarks for identifying tooth displacements while calculating their position alterations during the observation period. Validation processes involved comparing AI measurement results to the results obtained through manual evaluation.

## The primary outcomes analyzed included:

## Accuracy:

Define as the degree of agreement between AI predictions and manual measurements. The time demand between AI-based examination and standard manual testing represented one outcome while accuracy made up another outcome set. The reliability assessment of AI predictions required orthodontists to provide their ratings by choosing levels from 1 to 5 on a Likert scale. The analysis was performed using SPSS as the software platform. The connection between AI-based measurements and manual results was measured through Pearson's correlation coefficient. Bland-Altman plots showed how well the measurements matched each other regarding both bias and consistency level. Time efficiency analysis between the two methods involved the use of a paired t-test statistical procedure. The study used a p value of < 0.05 to determine statistical significance.

## Results:

Deriving its results from tooth movement assessments the AI model proved effective at 92% accuracy. AI-based measurements produced a mean absolute error which equalled 0.25 mm (±0.12 mm) when compared to manual analysis. The results indicate that AI-based assessments and manual ones share a strong positive correlation of r = 0.94 at p < 0.001 (Table 1). Through automation the AI system cut down the duration needed for performing measurement analysis. Analysis of image sets through AI-based systems took 3 seconds on average while manual assessment of each case needed 7 minutes (p < 0.001). AI-assisted measurements processed data at a point that equated to 98% faster than traditional approaches according to Table 2 results. Orthodontists assessed AI system reliability at 4.7 points out of 5 on the Likert scale because they showed great trust in its measurement accuracy. Research participants expressed a preference for AI-monitoring services over traditional manual assessment because they found them more efficient and convenient according to 86% of respondents in (Table 3). The findings indicate that AI-based automated image analysis provides a highly accurate, time-efficient, and user-preferred method for tracking orthodontic tooth movement (Table 1, Table 2 and Table 3).

## Discussion:

Artificial intelligence automation for image analysis presents a dependable system to monitor orthodontic tooth movement (OTM). Testing revealed that AI model precision for detecting changes in position reached 92% accuracy and its findings established a robust. Relationship (r = 0.94, p < 0.001) to manual assessment results. The research findings demonstrate that shallow learning algorithms help achieve effective dental image processing solutions as previously shown in scientific literature [1, 2]. The assessment of orthodontic tooth movement (OTM) continues to rely on digital caliper measurements of plaster models which serve as the current standard for manual analysis. The approach faces limitations from inconsistent reader interpretations although it demands major time commitment and substantial labor [3]. Research has confirmed that orthodontic measurements conducted manually show inconsistency because of variations in expert level and measurement environment factors [4, 5]. This study demonstrated that the AI model operated at a speed of 3 seconds per image set exceeding manual assessments that required 7 minutes which validated its time-saving capabilities [6]. The analysis of images using artificial intelligence both minimizes observer subjectivity and generates uniform measurements thus establishing itself as a valuable solution for clinical applications [7]. The AI algorithm performed with a mean absolute error at 0.25 millimeters that falls inside clinical tolerance levels. The results matched previously established deep learning standards because image processing systems measured landmarks and teeth segments with precision [8, 9]. Studies have confirmed that AI-powered methods execute human examiners' tasks with higher consistency and repeatability levels [10]. Machine learning, a subfield of AI, enables computers to identify and recognize patterns within large datasets, allowing for automated discovery and analysis [11].

The development of artificial intelligence with deep learning capabilities enables potential integration of automatic orthodontic tooth movement analysis systems for orthodontic clinical practice. By using AI-based systems healthcare providers can detect treatment deviations at early stages thereby enabling them to make necessary adjustment changes [12]. Through real-time AI monitoring clinicians can supervise orthodontic patients at a distance which offers improved patient care in tele-orthodontics conditions [13]. Despite skepticism within the orthodontic community toward tele-orthodontics and AI-driven treatments, these technologies are poised to shape the future of orthodontics [14]. Future research needs to embrace studies about using AI technology together with three-dimensional imaging solutions like cone-beam computed tomography (CBCT) to enable more accurate treatment of intricate cases [15]. Although effective in this research the AI system used in analysis demonstrates certain key limitations. The model trained on a particular dataset has restrictions in applicability when dealing with various clinical contexts. The accuracy might be influenced by different lighting conditions as well as image quality variations together with factors that are specific to individual patients. Two-dimensional photographs used by the AI model did not effectively display subtle movements of teeth in occlusal and buccolingual directions. Using AI together with 3D intraoral scanning systems would enhance both accuracy and clinical usage potential.

## Conclusion:

An AI-powered automated image analysis system achieved high measurement precision while obtaining strong relationships with manual assessments and performing efficiently for monitoring orthodontic tooth movement. Thus, AI image analytical technology should be adopted by orthodontists for better treatment along with standardized practices. Hence, merging new AI developments will lead to better precision and easier application of real-time orthodontic evaluations.

## Figures and Tables

**Table 1:** Accuracy of AI-based and manual measurements

**Measurement Method**	**Mean Tooth Movement (mm)**	**Mean Absolute Error (mm)**	**Correlation (r)**	**p-value**
AI-Based Analysis	1.75 ± 0.21	0.25± 0.12	0.94	<0.001
Manual Measurement	1.80 ± 0.19	-	-	-

**Table 2:** Time efficiency comparison between AI and manual methods

**Measurement Method**	**Time per Case**	**Time Reduction [%]**	**p-value**
AI-Based Analysis	3 sec	98%	<0.001
Manual Measurement	7 min	-	-

**Table 3:** Reliability and user preference for AI system

**Parameter**	**AI System Score 9 (Mean ± SD)**	**Preference (%)**
Reliability (Likert Scale)	4.7 ± 0.3	-
AI Preference (Users)	-	86%
